# The potential toxic impact of different gadolinium-based contrast agents combined with 7-T MRI on isolated human lymphocytes

**DOI:** 10.1186/s41747-018-0069-y

**Published:** 2018-11-28

**Authors:** Björn Friebe, Frank Godenschweger, Mahsa Fatahi, Oliver Speck, Dirk Roggenbuck, Dirk Reinhold, Annika Reddig

**Affiliations:** 1 Department of Radiology and Nuclear Medicine, Otto von Guericke University Magdeburg, 39120 Magdeburg, Germany; 2 Department of Biomedical Magnetic Resonance, Otto von Guericke University Magdeburg, 39120 Magdeburg, Germany; 3 Leibniz Institute for Neurobiology, 39118 Magdeburg, Germany; 4 Center for Behavioral Brain Sciences, Otto von Guericke University Magdeburg, 39118 Magdeburg, Germany; 5 German Center for Neurodegenerative Disease, Site Magdeburg, 39120 Magdeburg, Germany; 6 Medipan GmbH, 15827 Dahlewitz, Berlin Germany; 7 Institute of Biotechnology, Brandenburg University of Technology Cottbus–Senftenberg, 01958 Senftenberg, Germany; 8 Institute of Molecular and Clinical Immunology, Otto von Guericke University Magdeburg, Leipziger Str. 44, 39120 Magdeburg, Germany

**Keywords:** Apoptosis, Contrast media, Lymphocytes, Magnetic resonance imaging, DNA damage

## Abstract

**Background:**

To investigate a potentially amplifying genotoxic or cytotoxic effect of different gadolinium-based contrast agents (GBCAs) in combination with ultra-high-field 7-T magnetic resonance imaging (MRI) exposure in separated human peripheral blood lymphocytes.

**Methods:**

This *in vitro* study was approved by the local ethics committee and written informed consent was obtained from all participants. Isolated lymphocytes from twelve healthy donors were incubated with gadobutrol, gadoterate meglumine, gadodiamide, gadopentetate dimeglumine, or gadoxetate either alone or combined with 7-T MRI (1 h). Deoxyribonucleic acid (DNA) double-strand breaks were assessed 15 min after MRI exposure by automated γH2AX foci quantification. Cytotoxicity was determined at later endpoints by Annexin V/propidium iodide apoptosis assay (24 h) and [^3^H]-thymidine proliferation test (72 h). As a reference, lymphocytes from four different donors were exposed analogously to iodinated contrast agents (iomeprol, iopromide) in combination with computed tomography.

**Results:**

Baseline γH2AX levels (0.08 ± 0.02 foci/cell) were not significantly (*p* between 0.135 and 1.000) enhanced after administration of GBCAs regardless of MRI exposure. In contrast to the two investigated macrocyclic GBCAs, lymphocytes exposed to the three linear GBCAs showed a dose-dependent increase in apoptosis (maximum 186% of unexposed control, *p* < 0.001) and reduced proliferation rate (minimum 0.7% of unexposed control, *p* < 0.001). However, additional 7-T MRI co-exposure did not alter GBCA-induced cytotoxicity.

**Conclusions:**

Exposure of lymphocytes to different GBCAs did not reveal significant induction of γH2AX foci, and enhanced cytotoxicity was only observed in lymphocytes treated with the linear GBCAs used in this study, independent of additional 7-T MRI co-exposure.

**Electronic supplementary material:**

The online version of this article (10.1186/s41747-018-0069-y) contains supplementary material, which is available to authorized users.

## Key points

- No evidence of DNA damage after contrast-enhanced 7-T MRI *in vitro*.
- Cytotoxicity was only observed after incubation with the three investigated linear GBCAs.
- Cytotoxicity was not further enhanced after combined exposure to 7-T MRI.

## Background

As the number of magnetic resonance imaging (MRI) examinations and the applied field strength have been, and still are, increasing steadily, several questions about MRI safety as well as about the adverse effects of gadolinium-based contrast agents (GBCAs) have raised public concerns [1, 2]. Although the incidence of nephrogenic systemic fibrosis was strongly reduced by applying restrictive guidelines [3], new controversial data on the deposition of Gd in the brain and other tissues has accumulated [4–6].

Another major issue is whether electromagnetic fields applied in MRI can lead to genomic instability in humans. In recent years, several studies have investigated the genotoxic impact of MRI exposure on lymphocytes *in vivo* and *in vitro*, as reviewed in detail elsewhere [7–10]. While some authors reported enhanced Deoxyribonucleic acid (DNA) damage in human lymphocytes after MRI examination [11–14], others could not confirm these findings [15–19]. Until now, no clear evidence or mechanism about MRI-induced DNA lesions could be described. Furthermore, most studies did not compare unenhanced and contrast-enhanced MRI exposure. Reports investigating radiation damage after computed tomography (CT) demonstrated an amplification of DNA lesions in the presence of iodinated contrast agent (ICA), probably due to enhanced photoelectric absorption of x-rays by injected iodine atoms [20–23]. But whether GBCAs also alter a potential toxic effect induced by MRI or *vice versa* needs to be investigated in greater detail. An *in vivo* study by Yildiz et al. [24] revealed no significant increase in DNA damage in patients after uenhanced MRI, but instead a significant induction of DNA lesions when MRI exposure was combined with injection of non-ionic, linear gadodiamide. In contrast, no alterations in DNA double-strand break (DSB) formation assessed by γH2AX immunofluorescence microscopy were found by an *in vivo* study by Reddig et al. [19], regardless of administration of the macrocyclic GBCA gadobutrol. Nevertheless, studies investigating the impact of different kinds of GBCAs on lymphocyte toxicity, as well as data about the genotoxic or cytotoxic impact of various GBCAs in combination with ultra-high-field MRI, are rare. We applied ultra-high-field MRI to the potential effects, as previous publications [11–19] have been contradictory regarding the induction of biological effects.

Therefore, the aim of this *in vitro* study was to investigate a potentially amplifying effect on γH2AX foci formation or cytotoxicity induced by different classes of GBCAs in combination with ultra-high-field MRI exposure at 7 T in human peripheral blood lymphocytes.

## Methods

### Study design and participants

This *in vitro* study was approved by the local Ethics Committee (RAD 244) and written informed consent was obtained from all participants. Heparinised blood samples were collected from twelve healthy subjects (six women, six men; mean age 35.8 years) on the day of examination. Each donor sample was subdivided and finally incubated with seven different agents (five GBCAs, Mannitol 780 and Mannitol 1960) in two different concentrations, plus a positive and a negative control (16 samples per donor). These samples were divided into one group that was exposed to 7-T MRI and another group that was not exposed to 7-T MRI, resulting in 32 different experiments for each of the twelve donors (see below).

### Lymphocyte isolation

Peripheral blood mononuclear cells, here referred to as lymphocytes, were separated by density gradient centrifugation and suspended in standard RPMI-1640 medium supplemented with 10% fetal calf serum, 100 U/mL penicillin and 100 μg/mL streptomycin before MRI/CT scan, thereby enabling immediate assessment of DNA damage and lymphocyte activation after exposure.

### Contrast media and exposure conditions

As different chemical structures of GBCA are considered to have different risk profiles regarding nephrogenic systemic fibrosis and Gd deposition [4–6], we evaluated all four structural classes of GBCA (linear/macrocyclic; ionic/non-ionic). Five different GBCAs (Table 1) that were available in our department were utilised as ready-to-use solutions for injection as applied in clinics, in order to study a setting that is as close to a realistic clinical setting as possible. MRI contrast agents were applied at a physiological concentration of 2 mM, estimated assuming a recommended diagnostic dose in patients of 0.1 mmol GBCA per kilogram body weight distributed in 60–80 mL blood per kilogram body weight [25], as well as a tenfold higher dosage of 20 mM. Mannitol solutions, adjusted to lowest (gadodiamide; mannitol-780 mOsm/kg H_2_O) and highest (gadopentetate dimeglumine; mannitol-1960 mOsm/kg H_2_O) final osmolality of applied contrast agents, served as controls.

**Table 1:** List of contrast agents applied in this *in vitro* study

Imaging system	Chemical name	Trade name (manufacturer)	Structure, ionicity	Initial concentration	Osmolality (mOsm/kg H_2_O)	Viscosity (mPa·s, 37 °C)	Clearance
MRI	Gadobutrol	Gadovist (Bayer Vital, Leverkusen, Germany)	Macrocyclic, nonionic	1.0 mmol/mL	1603	5.0	Renal
MRI	Gadoterate meglumine	Dotarem (Guerbet, Roissy, France)	Macrocyclic, ionic	0.5 mmol/mL	1350	2.0	Renal
MRI	Gadodiamide	Omniscan (General Electric Healthcare Buchler, Braunschweig Germany)	Linear, nonionic	0.5 mmol/mL	780	1.4	Renal
MRI	Gadopentetate dimeglumine	Magnograf (Jenapharm, Jena, Germany)	Linear, ionic	0.5 mmol/mL	1960	2.9	Renal
MRI	Gadoxetate	Primovist (Bayer Pharma, Berlin, Germany)	Linear, ionic	0.25 mmol/mL	688	1.2	50% hepatic, 50% renal
CT	Iomeprol	Imeron 300 M (Bracco Imaging, Konstanz, Germany)	Monomeric, nonionic	300 mg iodine/mL	521	2.9	Renal
CT	Iopromide	Ultravist 300 (Bayer Vital, Leverkusen, Germany)	Monomeric, nonionic	300 mg iodine/mL	590	4.7	Renal

*MRI* magnetic resonance imaging, *CT* computed tomography

Lymphocytes and contrast agent were transferred into 96-well plates. Approximately 30 min after contrast agent administration one half of the samples was placed into the isocentre of a whole-body 7-T MRI scanner (Siemens Healthineers, Erlangen, Germany), whereas the other half was placed outside the magnet shielding at a distance of 8 m from the isocentre in a low magnetic field of approximately 50 mT, but within the scanning room to provide identical temperature conditions.

MRI exposure was performed in a 1-h scan procedure. To increase potential toxic effects, an echo planar imaging sequence was adjusted to the maximum permissible switched gradient and specific absorption rate, as described previously by Reddig et al. [17]. Twelve experiments with lymphocytes from different donors were performed.

As a control, analogously to MRI experiments, lymphocytes from four different donors were irradiated with x-rays by conducting a spiral CT scan (Aquilion Prime, Toshiba Medical Systems, Tustin, CA, USA). Here, two different ICAs were investigated (Table 1) at final doses of 5, 15, and 50 mg iodine per mL referring to previous studies [20, 23, 26]. Mannitol controls with the same osmolality as iopromid (590 mOsm/kg H_2_O) were included. To reach a high dose concentration of 50 mg iodine per mililitre with conventional contrast agent (300 mg I/mL), a large volume fraction of contrast agent solution needed to be added, representing one-sixth of the cell suspension.

After MRI/CT exposure, samples were transported to the laboratory within 10 min in a styrofoam box and processed immediately. Additionally, lymphocytes exposed prior to the MRI/CT scan with a DSB-inducing dose of 0.5 Gy γ-radiation or the apoptosis-inducing cytostatic drug camptothecin (CPT, 2 μM) were used as positive controls for the genotoxic and cytotoxicity assays, respectively.

### γH2AX immunofluorescence analysis

For genotoxicity assessment, γH2AX foci were quantified as a marker for DNA DSBs. Samples irradiated with 0.5 Gy γ-radiation served as positive controls. To allow phosphorylation after MRI/CT exposure, cell cultures were incubated in plates with 96 wells for an additional 15 min at 37 °C in 7% CO_2_. Afterwards, samples were washed in phosphate-buffered saline (PBS) and pipetted onto microscope slides. After fixation in 2% formaldehyde (15 min) and permeabilisation in 0.2% Triton X-100, cells were blocked in PBS containing 1% bovine serum albumin. Samples were incubated with an anti-phosphohistone H2AX mouse monoclonal IgG primary antibody (Millipore, Schwalbach, Germany; dilution 1:500) overnight at 4 °C. After washing in blocking buffer, slides were stained with a polyclonal goat anti-mouse IgG antibody conjugated to Alexa Fluor 488 (Lifetechnologies, Darmstadt, Germany; dilution 1:500) for 1 h at room temperature. Subsequently, slides were washed in PBS and covered with 4′6-diamidino-2-phenylindole-containing mounting medium. The amount of γH2AX foci was quantified from an average of 370 lymphocytes per sample by an automated digital microscopy system, based on z-stack images of five focal planes throughout each nucleus (AKLIDES; Medipan, Dahlewitz, Germany) [27].

### Apoptosis detection

An Annexin V/propidium iodide (PI) staining kit (Biolegend, San Diego, CA, USA) was used to assess cell viability 24 h after exposure. Samples simultaneously incubated with 2 μM CPT were used as positive controls. Lymphocytes were stained according to the manufacturer’s instructions and analysed by flow cytometry (BD LSRFortessa; BD Biosciences, Mountain View, CA, USA). A minimum of 15,000 lymphocytes per sample were classified. Since the rate of necrotic cells (PI^+^, Annexin V^−^) was below 1%, only the sum of early (PI^−^, Annexin V^+^) and late (PI^+^, Annexin V^+^) apoptotic cells was used for evaluation.

### Proliferation assay

A standard tritiated thymidine (^3^[H]-TdR) incorporation assay was used to analyse the level of DNA synthesis. In brief, lymphocytes had to be stimulated with 2 μg/mL phytohemagglutinin (PHA) directly after MRI/CT exposure. After 3 days the cells were pulsed with [^3^H]-TdR (0.2 μCi/well) for 6 h and subsequently harvested. For quantification the microplate liquid scintillation counter Wallac MicroBeta TriLux from Perkin Elmer was utilised (Waltham, MA, USA). Lymphocytes incubated with 2 μM CPT served as positive controls.

### Statistical analysis

Statistical analysis was performed by Graph Pad Prism software version 5.01 (Graph Pad Software, La Jolla, CA, USA) and all data in text and figures are presented as mean ± standard error of the mean. For comparison of negative control samples with multiple treatment conditions, significance levels were calculated by repeated measures ANOVA with a confidence interval of 95%, followed by Dunnett’s post hoc test. When single exposure conditions induced significant differences, direct comparison of matching samples with and without CT/MRI exposure was performed, using paired *t*-tests to detect potential additional or synergistic effects.

## Results

### Toxic impact of iodinated contrast agent during CT

**Fig. 1:**
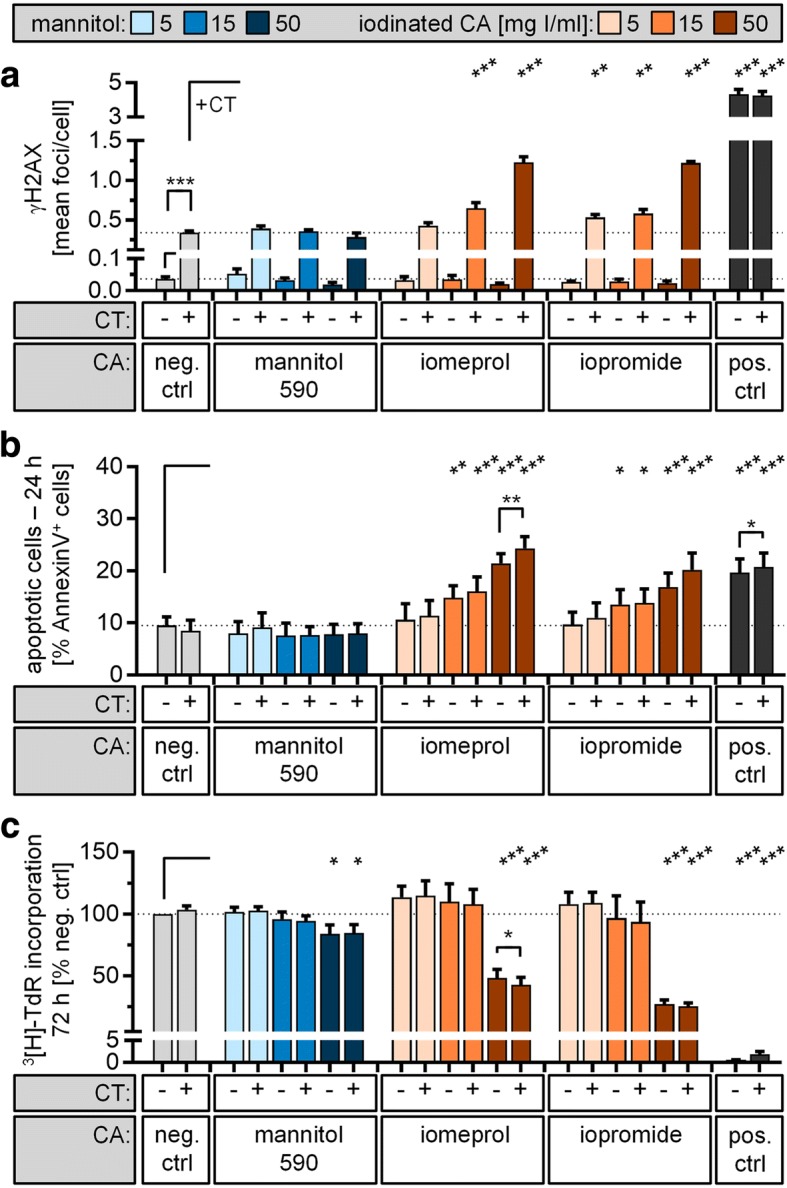
Genotoxic and cytotoxic impact of contrast-enhanced CT exposure. Isolated lymphocytes were incubated with indicated concentrations of iomeprol or iopromid. Samples incubated in cell culture medium only (*neg. ctrl*) or with mannitol solution with the same osmolality as iopromide (590 mOsm/kg H_2_O) served as negative controls. Additionally, samples were either irradiated by a thorax CT scan (*+*) or placed outside the CT scanner (-) at the same temperature. **a** The level of DNA double-strand breaks was assessed 15 min after CT exposure by γH2AX staining and automated foci quantification. Lymphocytes irradiated with 0.5 Gy served as positive controls (*pos. ctrl*). **b** Apoptosis rate was quantified after 24 h by Annexin V/propidium iodide staining combined with flow cytometric analysis. Samples incubated with 2 μM camptothecin served as positive controls. **c** For proliferation analysis, lymphocytes were activated with PHA directly after CT exposure. Level of DNA synthesis was determined after 72 h by [^3^H]-thymidine incorporation. Lymphocytes treated with 2 μM camptothecin served as positive controls. Diagrams displays mean ± standard error of the mean of four experiments (***p ≤ 0.001; **p ≤ 0.01; *p ≤ 0.05) and raw data are listed in Additional file 1: Tables S1–S3

To compare our data obtained from ultra-high-field MRI with exposure to ionising radiation and the published literature, we exposed lymphocytes to CT in the presence or absence of ICA at doses of 5, 15 and 50 mg iodine/mL as a control. As expected, CT exposure alone significantly increased the number of γH2AX foci from an initial 0.04 ± 0.01 (mean ± standard error of the mean) to 0.34 ± 0.02 foci per cell (Fig. 1a; Additional file 1: Table S1). The presence of ICA further enhanced γH2AX foci formation compared to CT exposure alone, with the strongest effect at the non-physiological high dose of 50 mg I/mL (iomeprol-50 + CT, 1.28 ± 0.06 foci/cell; iopromide-50 + CT, 1.23 ± 0.02 foci/cell; *p* < 0.001). In contrast, mannitol treatment or ICA without additional radiation did not alter baseline γH2AX levels significantly.

Determination of apoptotic cells 24 h after exposure revealed no significant differences in level of Annexin V^+^ cells between untreated (9.5 ± 1.6%) and CT exposed samples (8.5 ± 2.0%) (Fig. 1b; Additional file 1: Table S2). However, incubation of lymphocytes with ICA alone led to a dose-dependent induction of apoptosis, which was further increased for very high dose ICA (50 mg I/mL) in combination with CT exposure (iomeprol-50, 21.4 ± 1.9% vs iomeprol-50 + CT, 24.3 ± 2.3%, *p* = 0.008; iopromide-50, 16.9 ± 2.6% vs iopromide-50 + CT, 20.2 ± 3.2%, *p* = 0.099). Here, apoptosis rate was comparable to CPT-treated cells (CPT, 19.7 ± 2.6% vs CPT + CT, 20.8 ± 2.7%, *p* = 0.031).

A significant reduction in DNA synthesis was only observed at the highest applied dose of ICA (iomeprol-50, 48 ± 7%; iomeprol-50 + CT, 43 ± 6%; iopromide-50, 27 ± 3%; iopromide-50 + CT, 26 ± 3%; *p* < 0.001; Fig. 1c; Additional file 1: Table S3). As shown by paired *t*-tests, this effect was further significantly enhanced for iomeprol when lymphocytes were additionally irradiated by CT (*p* = 0.022). In contrast to the apoptosis assessment of unstimulated cells, CPT treatment affected proliferating cells much more strongly compared to high dose ICA, resulting in proliferation rates below the quantification limit (< 2%), and a high dose of mannitol inhibited lymphocyte proliferation (mannitol-50, 84 ± 7%, *p* = 0.016; mannitol-50 + CT, 85 ± 7%, *p* = 0.020), an effect which was probably due to the high amount of mannitol and volume fraction of one-sixth of the whole cell suspension (Fig. 1c; Additional file 1: Table S3).

### Toxic impact of gadolinium-based contrast agent during 7-T MRI

**Fig. 2:**
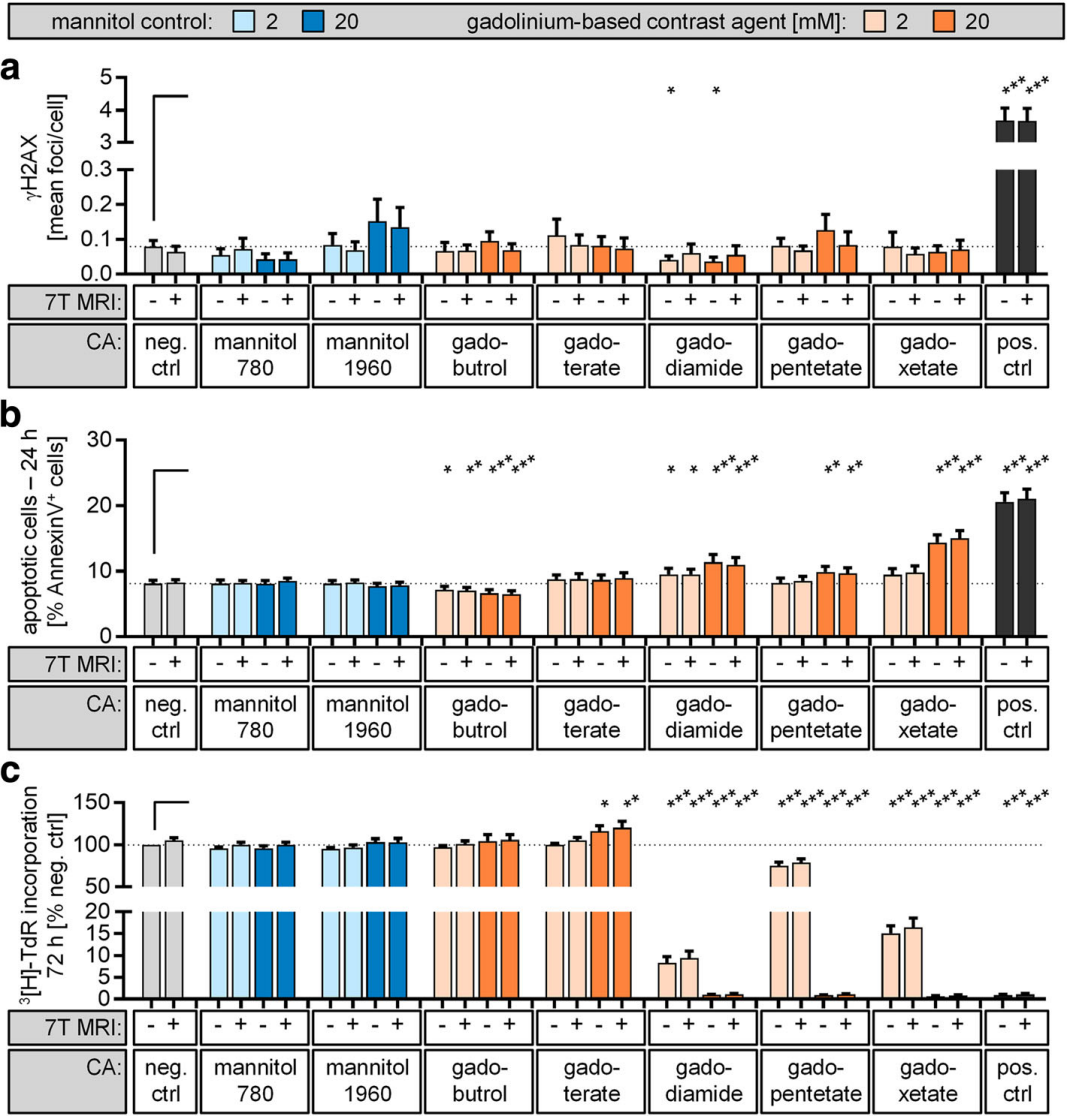
Genotoxic and cytotoxic impact of contrast-enhanced 7-T MRI exposure. Isolated lymphocytes were incubated with the indicated class and concentration of gadolinium-based contrast agent. Samples incubated in cell culture medium only (*neg. ctrl*) or with mannitol solution comparable with the lowest (780 mOsm/kg H_2_O; gadodiamide) and highest (1960 mOsm/kg H_2_O; gadopentetate dimeglumine) osmolality of GBCAs served as controls. Additionally, samples were either exposed to 7-T MRI (*+*) or placed outside the MRI scanner (-) at the same temperature. **a** The level of DNA double-strand breaks was assessed 15 min after exposure by γH2AX staining and automated foci quantification. Lymphocytes irradiated with 0.5 Gy served as positive controls (*pos. ctrl*). **b** Apoptosis rate was quantified after 24 h by Annexin V/propidium iodide staining combined with flow cytometric analysis. Samples treated with 2 μM camptothecin served as positive controls. **c** For proliferation analysis, lymphocytes were activated with PHA directly after MRI exposure. Level of DNA synthesis was determined after 72 h by [^3^H]-thymidine incorporation. Lymphocytes treated with 2 μM camptothecin served as positive controls. Diagrams display mean ± standard error of the mean of 12 experiments (***p ≤ 0.001; **p ≤ 0.01; *p ≤ 0.05) and raw data are listed in Additional file 1: Tables S4–S6

To investigate the impact of GBCAs on isolated lymphocytes, samples were incubated with different classes of GBCAs at a dose of 2 mM and 20 mM for 1 h with and without combined 7-T MRI exposure. Data revealed no significant induction of γH2AX foci (negative control, 0.08 ± 0.02 foci/cell) in any sample, independently of the GBCA structure, GBCA concentration or 7-T MRI exposure, whereas the number of DSBs was enhanced after γ-radiation (Fig. 2a; Additional file 1: Table S4). Surprisingly, lymphocytes treated with 2 mM or 20 mM gadodiamide showed a slight but significant reduction in γH2AX levels (gadodiamide-2 and -20, 0.04 ± 0.01 foci/cell, *p* = 0.030 and *p* = 0.012, respectively).

Assessment of apoptosis rate indicated reduced cell viability (*p* < 0.01) when lymphocytes were incubated with 2 mM gadodiamide as well as after treatment with 20 mM of the three linear GBCAs applied in this study (Fig. 2b; Additional file 1: Table S5). However, additional 7-T MRI exposure did not alter apoptosis rates compared to GBCA or CPT treatment alone. Statistical analysis even indicated a slight decrease in apoptosis when lymphocytes were cultured in the presence of the macrocyclic gadobutrol.

Compared to unexposed controls (100%), analysis of proliferation rates after 72 h of lymphocytes exposed to the three investigated linear GBCAs showed a strong, dose-dependent decrease in cell division, reaching levels below the quantification limit at concentrations of 20 mM, similar to positive CPT control (Fig. 2c; Additional file 1: Table S6). Whereas ICA and linear GBCAs induced apoptosis of unstimulated lymphocytes to a similar extent, DNA synthesis of the PHA-activated lymphocytes was inhibited more strongly by the linear GBCAs (gadodiamide, gadopentetate dimeglumine, gadoxetate) compared to applied ICA. However, a small but significant stimulatory effect was observed in activated lymphocytes incubated for 72 h with 20 mM of macrocyclic gadoterate.

## Discussion

Although MRI is considered a safe, non-invasive diagnostic imaging technique, in contrast to approaches based on ionising radiation exposure, several concerns about potential MRI-related health risks have arisen in the past few years and have not yet been sufficiently clarified to date. As a potential genotoxic effect of MRI would have a large impact on the clinical practice of MRI, several studies have been conducted in the past decade. Whereas some studies did not find any significant increase in genetic damage after MRI exposure, other studies reported a significant genotoxic MRI-related effect [11–19, 24, 28]. These studies, however, show a large diversity regarding field strengths, exposure parameters, and types of genotoxicity analysis. In addition, no established hypothesis for a potential mechanism can explain the results of the studies where significant effects were found.

Furthermore, it is known that no single genotoxic endpoint (*e.g.*, single/double-stand breaks, chromosomal aberrations, micronuclei) allows one to determine the carcinogenic risk of an agent. Consequently, further studies that try to investigate possible underlying mechanisms of genotoxicity should divide one sample into multiple aliquots in order to investigate several end-points of genotoxicity and cytotoxicity at the same time [7]. In this context, researchers also have to look for other possible genotoxic endpoints, such as oxidative stress, as has been reported by Erdamar et al. in 2014 [29].

As two studies that observed DNA damage were conducted after contrast-enhanced MRI [13, 24], a potential cause could also lie in a possible interaction mechanism with contrast media or in an interaction of contrast media with MRI. Therefore, in this study we examined the genotoxic and cytotoxic impact of different GBCAs on human blood lymphocytes and analysed a potential additive or synergistic effect when simultaneously exposed to 7-T MRI.

At our institution, ultra-high-field MRI is applied for research purposes only and administration of contrast agents is prohibited in studies conducted *in vivo* on humans. Thus, the current investigations had to be performed under *in vitro* conditions. However, the use of isolated, unshielded lymphocytes, the constant presence of high GBCA concentrations and the application of 7-T MRI combined with the maximum permissible switched gradient and specific absorption rate all present enhanced exposure conditions in these samples compared to *in vivo* examinations, and would presumably also maximised the level of cell damage and detectability.

Genotoxicity was determined by γH2AX staining, which is one of the most sensitive biomarkers for DNA DSB assessment. Our experiments confirmed the induction of γH2AX foci after CT exposure and an additional dose-dependent increase in the presence of ICA, while ICA alone did not lead to γH2AX formation. As has also been shown in previous publications [20–22], the extent of DNA damage was dependent on irradiation dose as well as on ICA concentration. DSB induced by unenhanced CT were mainly repaired and did not alter apoptosis or proliferation rate, whereas ICA-induced cytotoxicity was partially enhanced for high dose ICA in combination with CT. In contrast, GBCA treatment alone and simultaneous 7-T MRI exposure showed no evidence of enhanced γH2AX levels in peripheral lymphocytes. While Fiechter et al. [13] reported a significant increase in γH2AX after gadobutrol-enhanced MRI exposure, our current results support the findings of Brandt et al. [16] and Reddig et al. [19], who reported no induction of γH2AX foci in patients 5–30 min after contrast-enhanced MRI.

As Gd- and GBCA-induced cytotoxicity has been described for various cell types [5, 26, 30], we determined the level of cell death induced by different structural classes of GBCAs and investigated a potentially enhancing effect in combination with 7-T MRI. The apoptosis rate of lymphocytes was analysed 24 h after MRI exposure while GBCAs were still present. At a dose of 2 mM, a significant increase in cell death was only observed for the investigated linear, non-ionic gadodiamide, while a concentration of 20 mM induced apoptosis for all three linear GBCAs. This cytotoxic effect occurred independently of MRI exposure and was detectable to a stronger extent by the level of DNA synthesis determined after 72 h in activated lymphocytes. At a dose of 20 mM, analysed classes of linear GBCAs led to a strong reduction in the proliferation rate, whereas no decrease was observed for the two macrocyclic GBCAs included in our study. However, additional markers need to be assessed to clarify this observation in activated lymphocytes.

A surprising finding was a slight decrease in apoptosis when lymphocytes were cultured in the presence of the macrocyclic gadobutrol (Fig. 2b). This decrease seems difficult to explain, as no mechanism for potential MRI-induced primary or secondary genotoxic or cytotoxic effects is known. To investigate this effect more deeply in further studies, it could be helpful to perform additional apoptosis assays, such as caspase 3 activity, which was not part of this study.

Furthermore, the exposure conditions required for cytotoxicity assessment in our *in vitro* experiments differed in comparision to GBCA pharmacokinetics *in vivo*. Upon injection *in vivo* GBCA rapidly distributes to the extracellular space and is eliminated from the blood with a half-life of approximately 1.5 h in subjects with normal renal function [31, 32]. Therefore, the drawing of reliable conclusions from our *in vitro* data for clinical routines is difficult.

As shown by Cho et al. [30], exposure of human lymphocytes to non-chelated GdCl_3_ led to a dose-dependent induction of apoptosis and DNA damage, which were further enhanced when samples were co-exposed to Gd and an extremely low-frequency electromagnetic field. Our cytotoxicity data compare well with previously described dissociation characteristics of toxic free Gd^3+^ from chemically different GBCA chelates. In general, macrocyclic GBCAs are described as being more stable than linear compounds [5, 33]. Analysing the Gd^3+^ release of multiple GBCAs in human serum over a period of 15 days, Frenzel et al. [34] observed an increase of free Gd^3+^ from ionic and especially non-ionic linear GBCAs, whereas all macrocyclic GBCAs remained stable. A correlation between osmolality of GBCA and cytotoxicity, as described for porcine renal epithelial cells by Heinrich et al. [26], was not observed in our study. Our results confirmed increased toxicity induced by the group of investigated linear GBCAs compared to the two analysed macrocyclic ones. Nevertheless, further *in vitro* and *in vivo* studies are needed to evaluate all commercially available MRI contrast agents and to detect possible mechanisms of toxicity or potential interaction in combination with high- and ultra-high-field MRI.

Limitations of this study include the circumstances that only a limited amount of analyses could be conducted within this experimental setup, and no conclusions regarding different cell types, different DNA lesions or long-term effects *in vivo* can be drawn. Furthermore, effects due to ingredients other than the pure substance of the GBCA cannot be fully excluded. Since no exact mechanisms about potential MRI-induced primary or secondary genotoxic or cytotoxic effects are known, it is difficult to choose the most sensitive assay and best point in time. Until now, experimental procedures are mainly motivated by studies investigating ionising radiation. The exposure conditions required for cytotoxicity assessment in our *in vitro* experiments differed regarding GBCA pharmacokinetics *in vivo*, as mentioned above. Therefore, the genotoxic and cytotoxic impact of different GBCAs in MRI needs to be further examined, in particular regarding Gd tissue retention and possible pathological effects, especially under more reliable *in vivo* conditions.

In conclusion, our *in vitro* data demonstrate no induction of γH2AX foci in isolated human lymphocytes after contrast-enhanced 7-T MRI exposure with respect to the five investigated GBCAs. Enhanced cytotoxicity was observed for the three tested linear GBCAs compared to the two macrocyclic ones, but no further amplified cell damage was determined when GBCA exposure was combined with 7-T MRI.

## Additional file

Additional file 1: **Table S1.** Raw data for Fig. 1a, γH2AX level after native and contrast-enhanced CT exposure *in vitro*. **Table S2.** Raw data for Fig. 1b, apoptosis rate after native and contrast-enhanced CT exposure *in vitro*. **Table S3.** Raw data for Fig. 1c, proliferation rate after native and contrast-enhanced CT exposure *in vitro*. **Table S4.** Raw data for Fig. 2a, γH2AX level after native and contrast-enhanced 7-T magnetic resonance (MR) exposure *in vitro*. **Table S5.** Raw data for Fig. 2b, apoptosis rate after native and contrast-enhanced 7-T MR exposure *in vitro*. **Table S6.** Raw data for Fig. 2c, proliferation rate after native and contrast-enhanced 7-T MR exposure *in vitro*. (DOCX 63 kb)

## Abbreviations

^3^[H]-TdR: Tritiated thymidine
CPT: Camptothecin
CT: Computed tomography
DSB: Double-strand break
GBCA: Gadolinium-based contrast agent
ICA: Iodinated contrast agent
MRI: Magnetic resonance imaging
PBS: Phosphate-buffered saline
PHA: Phytohemagglutinin
PI: Propidium iodide
γH2AX: Phosphorylated histone H2AX
