# Supplementary material for: The potential toxic impact of different gadolinium-based contrast agents combined with 7-T MRI on isolated human lymphocytes
Source: Eur Radiol Exp. 2018 Nov 28;2:40. doi: 10.1186/s41747-018-0069-y (PMC6258802; doi:10.1186/s41747-018-0069-y)
Supplement: Supplementary file 1 — Table S1. Raw data for Fig. 1a, γH2AX level after native and contrast-enhanced CT exposure in vitro. Table S2. Raw data for Fig. 1b, apoptosis rate after native and contrast-enhanced CT exposure in vitro. Table S3. Raw data for Fig. 1c, proliferation rate after native and contrast-enhanced CT exposure in vitro. Table S4. Raw data for Fig. 2a, γH2AX level after native and contrast-enhanced 7-T magnetic resonance (MR) exposure in vitro. Table S5. Raw data for Fig. 2b, apoptosis rate after native and contrast-enhanced 7-T MR exposure in vitro. Table S6. Raw data for Fig. 2c, proliferation rate after native and contrast-enhanced 7-T MR exposure in vitro. (DOCX 63 kb) [file 41747_2018_69_MOESM1_ESM.docx]

Table S1: Raw data – Figure 1a, γH2AX level after native and contrast-enhanced CT exposure in vitro

| **Treatment** | **Concentration** | **CT** | **γH2AX [mean foci/cell]** | | | |
| --- | --- | --- | --- | --- | --- | --- |
|  |  |  | **#1** | **#2** | **#3** | **#4** |
| **neg. control** | **---** | **–** | 0,05 | 0,03 | 0,05 | 0,03 |
|  |  | **+** | 0,40 | 0,30 | 0,34 | 0,33 |
| **Mannitol – 590** | **5** | **–** | 0,05 | 0,09 | 0,06 | 0,02 |
|  |  | **+** | 0,43 | 0,45 | 0,40 | 0,29 |
|  | **15** | **–** | 0,03 | 0,04 | 0,05 | 0,01 |
|  |  | **+** | 0,32 | 0,42 | 0,35 | 0,34 |
|  | **50** | **–** | 0,03 | 0,02 | 0,03 | 0,00 |
|  |  | **+** | 0,32 | 0,39 | 0,29 | 0,14 |
| **Iomeprol**  **[mg I/ml]** | **5** | **–** | 0,07 | 0,02 | 0,02 | 0,02 |
|  |  | **+** | 0,54 | 0,40 | 0,43 | 0,34 |
|  | **15** | **–** | 0,07 | 0,02 | 0,05 | 0,01 |
|  |  | **+** | 0,73 | 0,60 | 0,79 | 0,48 |
|  | **50** | **–** | 0,03 | 0,02 | 0,02 | 0,02 |
|  |  | **+** | 1,38 | 1,16 | 1,30 | 1,07 |
| **Iopromide**  **[mg I/ml]** | **5** | **–** | 0,04 | 0,03 | 0,03 | 0,02 |
|  |  | **+** | 0,45 | 0,61 | 0,57 | 0,53 |
|  | **15** | **–** | 0,03 | 0,04 | 0,04 | 0,01 |
|  |  | **+** | 0,44 | 0,70 | 0,64 | 0,55 |
|  | **50** | **–** | 0,02 | 0,04 | 0,03 | 0,00 |
|  |  | **+** | 1,19 | 1,21 | 1,28 | 1,20 |
| **pos. control  (0.5 Gy)** | **---** | **–** | 4,78 | 4,05 | 4,83 | 3,66 |
|  |  | **+** | 4,51 | 3,55 | 4,44 | 4,50 |

Table S2: Raw data – Figure 1b: apoptosis rate after native and contrast-enhanced CT exposure in vitro

| **Treatment** | **Concentration** | **CT** | **Apoptotic cells [%]** | | | |
| --- | --- | --- | --- | --- | --- | --- |
|  |  |  | **#1** | **#2** | **#3** | **#4** |
| **neg. control** | **---** | **–** | 9,8 | 7,7 | 6,6 | 14,0 |
|  |  | **+** | 5,6 | 5,7 | 8,5 | 14,3 |
| **Mannitol – 590** | **5** | **–** | 5,3 | 6,5 | 5,5 | 14,7 |
|  |  | **+** | 6,7 | 5,5 | 6,8 | 17,5 |
|  | **15** | **–** | 5,7 | 4,5 | 5,6 | 14,6 |
|  |  | **+** | 6,0 | 5,6 | 6,7 | 12,4 |
|  | **50** | **–** | 6,7 | 5,0 | 6,1 | 13,5 |
|  |  | **+** | 6,2 | 5,0 | 7,4 | 13,4 |
| **Iomeprol**  **[mg I/ml]** | **5** | **–** | 6,3 | 5,7 | 11,4 | 19,0 |
|  |  | **+** | 6,4 | 8,4 | 11,0 | 19,7 |
|  | **15** | **–** | 12,9 | 10,6 | 14,6 | 21,3 |
|  |  | **+** | 11,2 | 12,7 | 16,5 | 23,8 |
|  | **50** | **–** | 19,2 | 18,5 | 21,3 | 26,7 |
|  |  | **+** | 21,7 | 20,8 | 23,8 | 30,9 |
| **Iopromide**  **[mg I/ml]** | **5** | **–** | 6,5 | 7,2 | 8,3 | 16,7 |
|  |  | **+** | 6,9 | 6,6 | 11,3 | 19,1 |
|  | **15** | **–** | 8,6 | 10,2 | 13,9 | 21,5 |
|  |  | **+** | 10,3 | 10,3 | 12,9 | 21,8 |
|  | **50** | **–** | 12,7 | 14,8 | 15,6 | 24,6 |
|  |  | **+** | 15,6 | 14,6 | 22,1 | 28,5 |
| **pos. control  (CPT – 2 µM)** | **---** | **–** | 18,4 | 16,9 | 16,1 | 27,4 |
|  |  | **+** | 18,7 | 18,5 | 17,2 | 28,7 |

Table S3: Raw data – Figure 1c, proliferation rate after native and contrast-enhanced CT exposure in vitro

| **Treatment** | **Concentration** | **CT** | **^3^[H]-TdR incorporation [% ctrl]** | | | |
| --- | --- | --- | --- | --- | --- | --- |
|  |  |  | **#1** | **#2** | **#3** | **#4** |
| **neg. control** | **---** | **–** | 100,0 | 100,0 | 100,0 | 100,0 |
|  |  | **+** | 104,8 | 99,9 | 111,6 | 97,9 |
| **Mannitol – 590** | **5** | **–** | 112,8 | 99,6 | 96,6 | 97,5 |
|  |  | **+** | 104,1 | 96,0 | 111,1 | 98,9 |
|  | **15** | **–** | 111,1 | 98,4 | 87,7 | 86,6 |
|  |  | **+** | 107,1 | 89,6 | 90,5 | 89,5 |
|  | **50** | **–** | 104,6 | 79,3 | 72,9 | 79,8 |
|  |  | **+** | 104,9 | 79,1 | 75,3 | 79,6 |
| **Iomeprol**  **[mg I/ml]** | **5** | **–** | 88,4 | 117,4 | 117,5 | 131,2 |
|  |  | **+** | 83,8 | 134,7 | 107,5 | 132,9 |
|  | **15** | **–** | 71,3 | 135,6 | 104,2 | 128,5 |
|  |  | **+** | 80,2 | 131,6 | 95,0 | 124,6 |
|  | **50** | **–** | 31,1 | 56,7 | 44,0 | 61,7 |
|  |  | **+** | 28,8 | 49,9 | 35,5 | 56,3 |
| **Iopromide**  **[mg I/ml]** | **5** | **–** | 81,7 | 115,8 | 108,9 | 126,1 |
|  |  | **+** | 86,4 | 115,5 | 107,5 | 126,9 |
|  | **15** | **–** | 52,8 | 135,5 | 83,7 | 114,3 |
|  |  | **+** | 55,4 | 131,1 | 81,0 | 106,4 |
|  | **50** | **–** | 24,2 | 29,1 | 20,3 | 35,3 |
|  |  | **+** | 24,0 | 28,9 | 18,3 | 30,7 |
| **pos. control  (CPT – 2 µM)** | **---** | **–** | 0,8 | 0,6 | 0,3 | 0,5 |
|  |  | **+** | 2,6 | 3,4 | 0,5 | 0,7 |

Table S4: Raw data – Figure 2a, γH2AX level after native and contrast-enhanced 7 T MR exposure in vitro

| **Treatment** | **Concentration** | **MR** | **γH2AX [mean foci/cell]** | | | | | | | | | | | |
| --- | --- | --- | --- | --- | --- | --- | --- | --- | --- | --- | --- | --- | --- | --- |
|  |  |  | **#1** | **#2** | **#3** | **#4** | **#5** | **#6** | **#7** | **#8** | **#9** | **#10** | **#11** | **#12** |
| **neg. control** | **---** | **–** | 0,02 | 0,15 | 0,07 | 0,16 | 0,19 | 0,06 | 0,11 | 0,02 | 0,06 | 0,05 | 0,02 | 0,05 |
|  |  | **+** | 0,03 | 0,16 | 0,07 | 0,05 | 0,19 | 0,07 | 0,08 | 0,01 | 0,06 | 0,03 | 0,03 | 0,02 |
| **Mannitol –  780** | **2** | **–** | 0,06 | 0,11 | 0,05 | 0,02 | 0,24 | 0,03 | 0,02 | 0,02 | 0,02 | 0,02 | 0,03 | 0,03 |
|  |  | **+** | 0,03 | 0,29 | 0,06 | 0,01 | 0,31 | 0,04 | 0,01 | 0,02 | 0,03 | 0,01 | 0,04 | 0,02 |
|  | **20** | **–** | 0,02 | 0,13 | 0,08 | 0,01 | 0,17 | 0,03 | 0,04 | 0,01 | 0,03 | 0,01 | 0,00 | 0,00 |
|  |  | **+** | 0,00 | 0,19 | 0,03 | 0,01 | 0,15 | 0,04 | 0,01 | 0,02 | 0,06 | 0,00 | 0,02 | 0,00 |
| **Mannitol – 1960** | **2** | **–** | 0,01 | 0,07 | 0,09 | 0,07 | 0,44 | 0,08 | 0,07 | 0,03 | 0,06 | 0,03 | 0,02 | 0,05 |
|  |  | **+** | 0,01 | 0,09 | 0,07 | 0,02 | 0,31 | 0,10 | 0,03 | 0,03 | 0,10 | 0,03 | 0,01 | 0,03 |
|  | **20** | **–** | 0,04 | 0,30 | 0,28 | 0,09 | 0,78 | 0,06 | 0,04 | 0,03 | 0,06 | 0,11 | 0,03 | 0,02 |
|  |  | **+** | 0,02 | 0,17 | 0,23 | 0,02 | 0,71 | 0,07 | 0,04 | 0,01 | 0,04 | 0,04 | 0,05 | 0,23 |
| **Gadobutrol**  **[mM]** | **2** | **–** | 0,00 | 0,20 | 0,03 | 0,03 | 0,28 | 0,06 | 0,08 | 0,04 | 0,06 | 0,02 | 0,00 | 0,01 |
|  |  | **+** | 0,06 | 0,07 | 0,12 | 0,02 | 0,22 | 0,07 | 0,06 | 0,04 | 0,06 | 0,06 | 0,03 | 0,00 |
|  | **20** | **–** | 0,04 | 0,22 | 0,27 | 0,05 | 0,21 | 0,04 | 0,12 | 0,02 | 0,11 | 0,03 | 0,02 | 0,00 |
|  |  | **+** | 0,01 | 0,17 | 0,17 | 0,02 | 0,15 | 0,06 | 0,12 | 0,02 | 0,06 | 0,02 | 0,02 | 0,01 |
| **Gadoterate**  **[mM]** | **2** | **–** | 0,01 | 0,19 | 0,13 | 0,02 | 0,60 | 0,12 | 0,03 | 0,01 | 0,11 | 0,08 | 0,02 | 0,03 |
|  |  | **+** | 0,01 | 0,09 | 0,14 | 0,04 | 0,38 | 0,12 | 0,06 | 0,02 | 0,06 | 0,04 | 0,03 | 0,03 |
|  | **20** | **–** | 0,01 | 0,07 | 0,15 | 0,15 | 0,32 | 0,07 | 0,04 | 0,01 | 0,06 | 0,03 | 0,04 | 0,03 |
|  |  | **+** | 0,02 | 0,04 | 0,28 | 0,01 | 0,31 | 0,06 | 0,05 | 0,04 | 0,05 | 0,02 | 0,02 | 0,01 |
| **Gadodiamide**  **[mM]** | **2** | **–** | 0,03 | 0,11 | 0,06 | 0,04 | 0,13 | 0,01 | 0,01 | 0,01 | 0,02 | 0,01 | 0,04 | 0,03 |
|  |  | **+** | 0,01 | 0,17 | 0,04 | 0,01 | 0,31 | 0,03 | 0,03 | 0,02 | 0,02 | 0,01 | 0,05 | 0,02 |
|  | **20** | **–** | 0,02 | 0,10 | 0,05 | 0,02 | 0,15 | 0,02 | 0,02 | 0,03 | 0,01 | 0,00 | 0,01 | 0,00 |
|  |  | **+** | 0,02 | 0,22 | 0,06 | 0,01 | 0,28 | 0,03 | 0,01 | 0,01 | 0,02 | 0,00 | 0,01 | 0,01 |
| **Gadopentetate**  **[mM]** | **2** | **–** | 0,02 | 0,12 | 0,17 | 0,09 | 0,26 | 0,09 | 0,05 | 0,03 | 0,07 | 0,02 | 0,06 | 0,00 |
|  |  | **+** | 0,06 | 0,16 | 0,08 | 0,03 | 0,12 | 0,07 | 0,07 | 0,02 | 0,11 | 0,03 | 0,05 | 0,02 |
|  | **20** | **–** | 0,01 | 0,20 | 0,20 | 0,07 | 0,58 | 0,13 | 0,02 | 0,02 | 0,06 | 0,04 | 0,06 | 0,12 |
|  |  | **+** | 0,01 | 0,14 | 0,10 | 0,01 | 0,49 | 0,06 | 0,06 | 0,00 | 0,06 | 0,01 | 0,05 | 0,02 |
| **Gadoxetate**  **[mM]** | **2** | **–** | 0,02 | 0,13 | 0,11 | 0,02 | 0,52 | 0,02 | 0,05 | 0,02 | 0,04 | 0,01 | 0,01 | 0,00 |
|  |  | **+** | 0,03 | 0,15 | 0,12 | 0,03 | 0,19 | 0,04 | 0,04 | 0,02 | 0,06 | 0,01 | 0,01 | 0,00 |
|  | **20** | **–** | 0,04 | 0,06 | 0,13 | 0,18 | 0,16 | 0,06 | 0,02 | 0,02 | 0,07 | 0,00 | 0,03 | 0,00 |
|  |  | **+** | 0,02 | 0,09 | 0,24 | 0,06 | 0,28 | 0,04 | 0,04 | 0,02 | 0,05 | 0,00 | 0,00 | 0,00 |
| **pos. control  (0.5 Gy)** | **---** | **–** | 2,51 | 5,25 | 3,21 | 4,11 | 4,68 | 4,86 | 4,61 | 3,75 | 4,84 | 3,69 | 1,20 | 1,50 |
|  |  | **+** | 2,23 | 5,29 | 2,80 | 5,22 | 4,80 | 4,58 | 3,82 | 3,33 | 5,05 | 3,70 | 1,38 | 1,83 |

Table S5: Raw data – Figure 2b, apoptosis rate after native and contrast-enhanced 7 T MR exposure in vitro

| **Treatment** | **Concentration** | **MR** | **Apoptotic cells [%]** | | | | | | | | | | | |
| --- | --- | --- | --- | --- | --- | --- | --- | --- | --- | --- | --- | --- | --- | --- |
|  |  |  | **#1** | **#2** | **#3** | **#4** | **#5** | **#6** | **#7** | **#8** | **#9** | **#10** | **#11** | **#12** |
| **neg. control** | **---** | **–** | 7,5 | 10,6 | 8,0 | 8,2 | 5,9 | 7,3 | 8,3 | 5,6 | 8,8 | 5,8 | 10,9 | 10,3 |
|  |  | **+** | 7,6 | 9,7 | 7,6 | 8,3 | 6,4 | 8,5 | 9,0 | 6,0 | 8,8 | 5,9 | 10,9 | 10,3 |
| **Mannitol –  780** | **2** | **–** | 7,2 | 9,7 | 7,1 | 8,2 | 6,0 | 7,1 | 9,8 | 5,8 | 10,9 | 6,0 | 8,8 | 10,8 |
|  |  | **+** | 8,4 | 9,1 | 7,3 | 7,9 | 6,7 | 8,3 | 9,6 | 6,0 | 8,3 | 6,6 | 9,3 | 10,7 |
|  | **20** | **–** | 8,5 | 11,5 | 7,3 | 8,8 | 6,5 | 7,4 | 8,6 | 6,8 | 7,9 | 4,8 | 8,9 | 9,9 |
|  |  | **+** | 7,8 | 10,5 | 8,4 | 9,3 | 6,6 | 8,8 | 9,1 | 6,0 | 8,6 | 7,1 | 9,7 | 10,7 |
| **Mannitol – 1960** | **2** | **–** | 7,3 | 7,4 | 7,6 | 7,8 | 6,8 | 7,8 | 8,6 | 6,1 | 9,9 | 6,6 | 10,1 | 11,3 |
|  |  | **+** | 7,6 | 7,8 | 7,6 | 8,3 | 6,8 | 8,4 | 9,9 | 5,9 | 9,6 | 7,0 | 10,2 | 10,0 |
|  | **20** | **–** | 6,5 | 9,3 | 6,3 | 6,5 | 8,2 | 8,7 | 8,4 | 7,2 | 7,2 | 4,2 | 10,2 | 9,5 |
|  |  | **+** | 6,8 | 9,2 | 7,5 | 6,3 | 7,6 | 9,2 | 8,8 | 7,0 | 8,2 | 4,7 | 9,7 | 9,5 |
| **Gadobutrol**  **[mM]** | **2** | **–** | 4,6 | 7,3 | 6,7 | 6,4 | 5,8 | 7,3 | 9,3 | 6,2 | 7,8 | 4,1 | 9,8 | 10,2 |
|  |  | **+** | 5,2 | 6,7 | 5,7 | 6,2 | 6,3 | 7,0 | 8,8 | 5,8 | 7,4 | 4,9 | 10,5 | 9,6 |
|  | **20** | **–** | 5,3 | 6,5 | 5,1 | 5,9 | 5,6 | 6,5 | 8,9 | 7,5 | 5,5 | 3,5 | 9,9 | 9,2 |
|  |  | **+** | 5,3 | 6,5 | 4,7 | 6,2 | 5,7 | 7,1 | 8,3 | 6,5 | 6,6 | 3,2 | 9,0 | 9,0 |
| **Gadoterate**  **[mM]** | **2** | **–** | 7,3 | 12,2 | 6,7 | 7,6 | 7,1 | 7,9 | 10,1 | 6,3 | 9,8 | 6,1 | 11,8 | 12,0 |
|  |  | **+** | 8,1 | 10,6 | 7,1 | 8,0 | 6,2 | 7,7 | 11,0 | 6,5 | 9,2 | 4,5 | 14,9 | 11,8 |
|  | **20** | **–** | 7,5 | 8,7 | 4,6 | 6,5 | 7,5 | 9,3 | 10,9 | 7,1 | 10,3 | 6,8 | 14,2 | 10,8 |
|  |  | **+** | 7,1 | 8,2 | 4,5 | 6,8 | 7,1 | 9,6 | 11,9 | 7,2 | 12,0 | 6,9 | 13,9 | 12,0 |
| **Gadodiamide**  **[mM]** | **2** | **–** | 6,8 | 9,4 | 4,8 | 7,6 | 7,1 | 10,4 | 13,5 | 7,3 | 12,9 | 6,8 | 13,1 | 14,3 |
|  |  | **+** | 7,1 | 11,5 | 5,2 | 8,5 | 7,3 | 9,9 | 13,0 | 6,6 | 12,1 | 7,8 | 12,9 | 12,2 |
|  | **20** | **–** | 8,6 | 14,7 | 6,2 | 8,0 | 7,4 | 10,9 | 16,3 | 7,3 | 15,5 | 9,1 | 16,9 | 15,5 |
|  |  | **+** | 8,8 | 13,2 | 5,5 | 7,2 | 8,7 | 11,9 | 15,3 | 8,0 | 14,8 | 7,5 | 15,9 | 15,3 |
| **Gadopentetate**  **[mM]** | **2** | **–** | 6,6 | 7,4 | 5,5 | 7,5 | 6,7 | 8,3 | 11,8 | 6,3 | 10,1 | 4,0 | 11,4 | 12,6 |
|  |  | **+** | 6,5 | 9,4 | 6,9 | 7,0 | 6,8 | 8,4 | 11,3 | 6,8 | 9,2 | 5,6 | 11,1 | 13,4 |
|  | **20** | **–** | 8,8 | 9,7 | 4,8 | 7,1 | 7,9 | 11,8 | 13,6 | 8,5 | 12,5 | 7,1 | 13,6 | 13,1 |
|  |  | **+** | 9,1 | 9,7 | 5,5 | 7,0 | 7,8 | 10,8 | 13,6 | 7,4 | 13,0 | 5,7 | 13,5 | 12,9 |
| **Gadoxetate**  **[mM]** | **2** | **–** | 7,1 | 9,0 | 5,5 | 7,3 | 6,9 | 9,6 | 11,8 | 7,6 | 13,7 | 7,0 | 13,6 | 14,6 |
|  |  | **+** | 0,03 | 0,15 | 0,12 | 0,03 | 0,19 | 0,04 | 0,04 | 0,02 | 0,06 | 0,01 | 0,01 | 0,00 |
|  | **20** | **–** | 0,04 | 0,06 | 0,13 | 0,18 | 0,16 | 0,06 | 0,02 | 0,02 | 0,07 | 0,00 | 0,03 | 0,00 |
|  |  | **+** | 0,02 | 0,09 | 0,24 | 0,06 | 0,28 | 0,04 | 0,04 | 0,02 | 0,05 | 0,00 | 0,00 | 0,00 |
| **pos. control  (CPT – 2 µM)** | **---** | **–** | 2,51 | 5,25 | 3,21 | 4,11 | 4,68 | 4,86 | 4,61 | 3,75 | 4,84 | 3,69 | 1,20 | 1,50 |
|  |  | **+** | 2,23 | 5,29 | 2,80 | 5,22 | 4,80 | 4,58 | 3,82 | 3,33 | 5,05 | 3,70 | 1,38 | 1,83 |

Table S6: Raw data – Figure 2c, proliferation rate after native and contrast-enhanced 7 T MR exposure in vitro

| **Treatment** | **Concentration** | **MR** | **^3^[H]-TdR incorporation [% neg.control]** | | | | | | | | | | | |
| --- | --- | --- | --- | --- | --- | --- | --- | --- | --- | --- | --- | --- | --- | --- |
|  |  |  | **#1** | **#2** | **#3** | **#4** | **#5** | **#6** | **#7** | **#8** | **#9** | **#10** | **#11** | **#12** |
| **neg. control** | **---** | **–** | 100,0 | 100,0 | 100,0 | 100,0 | 100,0 | 100,0 | 100,0 | 100,0 | 100,0 | 100,0 | 100,0 | 100,0 |
|  |  | **+** | 102,8 | 92,1 | 99,3 | 126,9 | 89,9 | 112,1 | 120,9 | 110,3 | 103,7 | 101,2 | 95,1 | 107,3 |
| **Mannitol –  780** | **2** | **–** | 93,9 | 91,5 | 99,4 | 100,1 | 92,7 | 88,5 | 96,3 | 97,7 | 98,2 | 104,2 | 89,0 | 101,9 |
|  |  | **+** | 101,1 | 84,1 | 95,0 | 117,6 | 86,6 | 103,9 | 109,0 | 103,3 | 108,4 | 103,7 | 88,5 | 104,2 |
|  | **20** | **–** | 72,1 | 90,6 | 100,0 | 95,1 | 93,1 | 89,3 | 97,6 | 104,9 | 113,0 | 100,3 | 89,3 | 107,1 |
|  |  | **+** | 91,0 | 87,9 | 89,1 | 114,2 | 86,2 | 104,2 | 111,0 | 111,7 | 109,0 | 98,0 | 87,6 | 110,1 |
| **Mannitol – 1960** | **2** | **–** | 97,7 | 93,3 | 95,9 | 102,8 | 92,3 | 86,3 | 90,2 | 97,7 | 94,2 | 103,7 | 89,8 | 100,8 |
|  |  | **+** | 86,2 | 85,7 | 99,9 | 115,9 | 81,9 | 90,3 | 105,2 | 99,3 | 101,1 | 101,5 | 93,9 | 104,0 |
|  | **20** | **–** | 115,8 | 85,8 | 98,1 | 114,7 | 78,9 | 93,9 | 108,5 | 105,3 | 100,7 | 121,2 | 114,4 | 104,2 |
|  |  | **+** | 114,3 | 75,3 | 97,4 | 118,5 | 73,4 | 102,0 | 124,8 | 106,9 | 105,0 | 98,6 | 117,9 | 99,9 |
| **Gadobutrol**  **[mM]** | **2** | **–** | 102,5 | 89,8 | 94,4 | 105,4 | 103,2 | 95,0 | 102,2 | 102,8 | 88,9 | 84,3 | 100,5 | 99,7 |
|  |  | **+** | 104,0 | 82,5 | 99,8 | 115,9 | 94,5 | 107,4 | 116,5 | 114,3 | 77,7 | 101,5 | 97,8 | 101,8 |
|  | **20** | **–** | 142,2 | 77,0 | 82,8 | 114,3 | 95,7 | 76,7 | 100,7 | 89,8 | 92,1 | 147,6 | 143,4 | 91,2 |
|  |  | **+** | 131,3 | 76,1 | 84,8 | 119,5 | 85,8 | 92,7 | 115,0 | 97,0 | 106,6 | 137,4 | 129,8 | 94,2 |
| **Gadoterate**  **[mM]** | **2** | **–** | 100,0 | 90,2 | 99,0 | 109,6 | 98,3 | 96,0 | 97,0 | 104,4 | 103,5 | 99,9 | 96,5 | 108,5 |
|  |  | **+** | 102,6 | 85,5 | 95,1 | 120,6 | 93,7 | 107,4 | 111,5 | 118,7 | 109,4 | 113,7 | 96,8 | 110,1 |
|  | **20** | **–** | 146,7 | 87,7 | 96,9 | 130,2 | 111,1 | 102,1 | 96,1 | 95,6 | 119,7 | 159,8 | 138,1 | 104,6 |
|  |  | **+** | 138,1 | 87,8 | 89,0 | 128,6 | 98,6 | 113,6 | 121,0 | 108,9 | 122,9 | 181,6 | 146,4 | 105,0 |
| **Gadodiamide**  **[mM]** | **2** | **–** | 10,3 | 8,3 | 9,8 | 19,1 | 3,1 | 13,7 | 5,3 | 11,8 | 2,1 | 8,2 | 3,0 | 4,8 |
|  |  | **+** | 9,1 | 8,6 | 8,7 | 17,5 | 2,2 | 16,8 | 4,7 | 14,0 | 3,2 | 14,9 | 4,0 | 9,6 |
|  | **20** | **–** | 1,3 | 1,4 | 0,8 | 1,8 | 0,4 | 0,5 | 0,9 | 0,7 | 0,7 | 1,6 | 1,0 | 0,4 |
|  |  | **+** | 2,4 | 1,7 | 0,6 | 1,6 | 0,5 | 1,2 | 0,4 | 1,1 | 0,9 | 1,8 | 0,5 | 0,7 |
| **Gadopentetate**  **[mM]** | **2** | **–** | 99,0 | 60,0 | 57,1 | 75,2 | 80,1 | 64,1 | 86,1 | 57,7 | 79,6 | 81,3 | 100,2 | 60,7 |
|  |  | **+** | 93,8 | 55,3 | 58,1 | 73,5 | 77,6 | 79,4 | 100,7 | 61,1 | 91,7 | 98,7 | 93,4 | 62,5 |
|  | **20** | **–** | 1,5 | 1,2 | 0,6 | 1,3 | 0,5 | 0,7 | 1,1 | 0,8 | 0,5 | 1,3 | 0,7 | 0,7 |
|  |  | **+** | 1,9 | 1,5 | 0,7 | 1,4 | 0,7 | 1,4 | 0,7 | 1,2 | 0,7 | 1,2 | 1,1 | 0,7 |
| **Gadoxetate**  **[mM]** | **2** | **–** | 17,6 | 15,6 | 18,0 | 26,0 | 6,1 | 20,8 | 8,5 | 16,8 | 10,4 | 19,8 | 8,3 | 12,9 |
|  |  | **+** | 17,6 | 13,8 | 16,5 | 28,3 | 6,5 | 27,5 | 8,1 | 18,7 | 9,4 | 24,6 | 10,3 | 16,0 |
|  | **20** | **–** | 1,4 | 0,9 | 0,4 | 1,1 | 0,2 | 0,4 | 0,7 | 0,6 | 0,6 | 0,8 | 0,7 | 0,4 |
|  |  | **+** | 1,7 | 1,2 | 0,5 | 1,5 | 0,3 | 1,0 | 0,4 | 1,1 | 0,6 | 0,9 | 0,7 | 0,5 |
| **pos. control  (CPT – 2 µM)** | **---** | **–** | 2,0 | 1,3 | 0,6 | 1,7 | 0,3 | 0,6 | 0,7 | 0,7 | 0,9 | 1,7 | 0,6 | 0,3 |
|  |  | **+** | 2,4 | 1,9 | 0,5 | 2,0 | 0,3 | 1,3 | 0,5 | 1,3 | 1,4 | 0,9 | 0,5 | 0,5 |
